# Treatment of Eczema

**Published:** 1884-03

**Authors:** Henry J. Reynolds

**Affiliations:** Professor of Dermatology, College of Physicians and Surgeons, Chicago


### ARTICLE III.

TREATMENT OF ECZEMA. A Lecture by HENRY J. REYNOLDS, M.D., Professor of Dermatology, College of Physicians and Surgeons, Chicago. Reported by R. W. Mathers, of San Francisco, Cal., of the Senior Class.

When we consider the scores of names that have been made use of by the different authors to designate the numerous forms of eczema, as a natural consequence we get the idea that the treatment must necessarily be very varied in order to fullfil the numerous requirements resulting from so many different existing conditions.

When we consider, again, that many if not all these numerous forms of the disease are or may be produced, or at least aggravated, by external causes, we necessarily arrive at the conclusion that the treatment, in order to be the most beneficial, must be to a great extent *external* or local in character.

And again, a consideration of the fact that nearly all of these various manifestations of eczema tend to spontaneous recovery when the part is simply protected from external or local irritation, suggests the idea that the external or local treatment must be mainly conducted with a view to the removal and continuous suspension of all forms of external or local irritation.

The treatment then must be mainly external; its chief object being the removal and continuous exclusion of all irritation. In no disease of the human body, however, are careful observation, scientific investigation, good judgment, and common sense more essential in the physician than in the treatment of eczema in its various forms, locations, phases, and stages. It cannot be intelligently managed by rule or by any specific line of treatment; the remedies must be adapted to the particular existing conditions met with in each individual case. What would prove useful in one stage, in one form, in one locality or in one person, may be not only useless in another, but absolutely injurious. The secret of successful management of this disease, perhaps more than all others, lies not so much in the amount of knowledge the physician possesses, as it does in the proper and scientific application of what he does know. The pathology being absolutely identical in no two cases, so the treatment must necessarily vary; and a knowledge of specified lines of treatment, or certain combinations of drugs said to be useful, with a neglect of consideration of the principles upon which treatment should be based in each individual case, is liable to mislead.

With these few ideas constantly in mind, with the confidence and coöperation of the patient, with the possession of good judgment and common sense, with even a limited amount of scientific knowledge, the physician is prepared to intelligently undertake the management of each case as it presents itself, no matter what its particular form or name may be; and is in the majority of cases, under such circumstances, warranted in guaranteeing a cure.

In a disease which, untreated, naturally tends to chronicity it is of the utmost importance that in rendering a prognosis, the physician secure for himself an abundance of time, in which to complete the cure. The coöperation of the patient with regard, to the length of time he is willing to persist in the carrying out of the prescribed treatment, is as essential to the ultimate success of the same, as the remedies made use of. The physician is many times defeated in a cure in chronic cases solely by lack of persistence in the treatment on the part of the patient.

The hosts of remedies for the external treatment of eczema may be embraced under the heads of oleaginous preparations, dusting powders, and various astringent, alkaline and anodyne lotions.

Perhaps all cases presented for treatment may be intelligently managed by dividing them into three classes: First, those the features of which are mainly of an *acute* character; second, those forms wherein the predominating features are *subacute* in type; and third, that class of cases that take on the indolent or *chronic* form. The treatment may then be arranged accordingly; always subject, however, to constant variations according to conditions warranting the same; for instance, the acute may very soon take on a subacute form; the subacute may approximate in character the acute or the chronic, and the chronic may at any time, owing to circumstances perhaps not under the control of the physician, develop acute symptoms. Here, as before stated, the physician is called upon for common sense, good judgment, power of discrimination, etc., which are as essential elements in successful treatment as an unlimited knowledge of remedies.

First, then, before adopting any line of treatment, determine what the predominating features are; whether they be acute, subacute or chronic. To a naturally close observer, with even a limited amount of experience, this is a matter of no great difficulty.

*Acute.*—In this form of the disease, soothing measures usually give the best satisfaction. The effect of water and atmospheric action upon a surface of this kind seems usually to be injurious, and to fulfil the indications in this case, the different oleaginous preparations generally answer the best purpose. To envelop the part in a lotion of equal parts of oleum amygdalæ dulcis and aqua calcis, in certain cases, works admirably. The oleum olivæ or oleum lini may be substituted for the oleum amygdalæ. Various poultices answer as well or better in some instances. They may be made of linseed meal, ground slippery elm bark, etc., and in order to do the most good, should be applied hot or warm, and allowed to remain as long as possible without changing. By covering them with oiled silk, they will retain the heat longer, and thus obviate the necessity of so frequent changing.

*Subacute.*—In this class of cases, which may assume this form from the beginning, or may follow the acute, the various dusting powders, certain soothing and mildly stimulating ointments, etc., are useful. Lycopodium, carbonate of magnesia, bismuth, oxide of zinc, boracic acid, camphor, alkaline remedies in the form of soap, iodoform and salicylic acid, alone or in various combinations and proportions, or combined with certain mild unguents, as ung. aquæ rosæ, ung. petrolati, etc., will be found among the more useful of this class of remedies. For instance:—

℞ Zinci oxid.........................................ʒi–ii

Pulv. camph.......................................ʒss–i

Pulv. lycopodii...................................℥i

Sig.—Apply twice or three times a day, after cleansing with soap and hot water.

℞ Pulv. iodoformi..............................grs. x–xx
Zinci oxid........................................ʒi
Ung. aq. rosæ vel petrolati..............℥i
M. Sig.—Apply two or three times a day.

The camphor and oxide of zinc are particularly useful if an antipruritic effect be required. Soap in this stage should be only mildly alkaline, some of the hard or soda soaps being rather the preferable form. It should again be borne in mind that during the treatment of this stage the symptoms may at any time assume either the acute or chronic forms. and necessitate a variation of the treatment accordingly.

*Chronic.*—In this form, which may be chronic from the commencement, or may follow the preceding form, more active and stimulating measures are required. The soaps which, in this stage, are used for their stimulant and antipruritic effect, dissolving and getting rid of pathological products, which create irritation, induce pruritus, prevent physiological action, etc., should be of the strongly alkaline or potash variety, and should be freely used. The different tarry preparations seem to be among the more beneficial remedies in this stage. The application of clear oxide of zinc, boracic acid, etc., in powder, governed according to the particular condition of each case, answers a good purpose. If there be considerable exudation with a tendency to pus formation, and not much bright redness of the surface or acute symptoms, the application of clear boracic acid in fine powder, after washing with sapo viridis and hot water, twice a day, works admirably, especially so in eczema of the leg with venous varicosity and infiltration, the powder being then secured by application of a snug bandage, rubber, with loose cloths interposed between it and the leg being in most cases preferable. The tarry preparations may be used in the form of lotions or ointments, the latter, however, being preferable if much excoriation or fissures exist, in a manner similar to the following:

℞ Picis liquidae......................................℥i
Saponis viridis.........................................℥ii
Spts. lavandulæ....... ..............................ʒii
Spts. rect. ad..........................................℥viii
M. Sig.—Apply with friction three times a day.

℞ Olei cadini.. ............................................ʒi
Acid boracici ....... ...................................ʒi
Ung. aq. rosae vel ung. petrolati.................℥i
M. Sig.—Rub gently into the skin three times a day,

The pix liquida or the oleum betullæ albæ may be substituted for the oleum cadini.

It must be remembered that this chronic form may at any time develope acute symptoms, at which time the measures must not be so active; or when first presented for treatment, it may be chronic as regards duration and still the predominating symptoms be acute. Absolute cleanliness should be enjoined in all stages when practicable. In eczema of the scalp where the part is liable to be covered with crusts and scales, they should be first removed by soaking with oily preparations, washing, etc. The treatment may then be adapted to the condition as in other localities. Compression, by means of plasters, is useful in many chronic and subacute cases where the condition, or location, render the application of a bandage inadmissible.

*Constitutional.*—While it may be conceded that in the majority of cases the treatment must be mainly external in character, it is, nevertheless, essential that the general system be kept in a condition that will necessarily favor the desired reparative process. For this purpose, it is quite probable again, that no specific line of treatment can be laid down that may be suitable in each and every case. It is the custom of many physicians to prescribe arsenic in this disease, but instead of doing good it is, no doubt, absolutely injurious in many cases; this is especially true of the acute forms. Many of these forms seem to be influenced materially by malaria, and in this case the anti-malarial treatment should be adopted. The general condition of the skin, alimentary canal and other organs should be regulated, if necessary. Arsenic may be tried in the dry, or squamous varieties, if other measures fail. In the pustular or other forms, wherein a pyogenic tendency exists, the sulphide of calcium is well worthy of a trial and sometimes works admirably. It may be given in doses of from one-tenth to one grain three times a day.

